# Genome resequencing and transcriptome analysis reveal the molecular mechanism of albinism in *Cordyceps militaris*

**DOI:** 10.3389/fmicb.2023.1153153

**Published:** 2023-04-11

**Authors:** Ying Zhao, YuDong Liu, Xun Chen, Jun Xiao

**Affiliations:** ^1^Institute of Edible Fungi, Liaoning Academy of Agricultural Sciences, Shenyang, China; ^2^College of Agriculture, Shihezi University, Shihezi, China; ^3^Key Laboratory of Special Fruits and Vegetables Cultivation Physiology and Germplasm Resources Utilization of Xinjiang Production and Construction Crops, Shihezi University, Shihezi, China

**Keywords:** *Cordyceps militaris*, albinism, WCC, *CmPKS*, light signal

## Abstract

Light is an important regulator of most fungal life activities and transmits signals through certain photoreceptor proteins such as phytochromes and cryptochromes. However, the light response mechanism varies across different fungi. The WCC complex composed of white collar-1 (WC-1) and white collar-2 (WC-2) is considered to be the key factor regulating fungal albinism. The photoreceptor protein Vivid (VVD) is the negative regulator of the WCC complex. In this study, we discovered an albino mutant (*Alb*) generated by ^60^Co-γ-ray irradiation from *Cordyceps militaris* (*C. militaris*). This mutant showed albinism of the mycelia and fruiting bodies under light, and the fruiting bodies developed normally. However, this phenotype in *Alb* differed from that in the *CmWC-1* mutant. This suggests that *CmWC1* may not be mutated in *Alb*. A mutated polyketide synthase (*CmPKS*) was found through genome resequencing analysis. *CmPKS* was significantly induced by a light signal, and its mutation reduced melanin accumulation in *C. militaris*. In addition, we found that a zinc-finger domain-containing protein (CmWC-3) was induced by a light signal and interacted with CmWC-1 and CmVVD. Moreover, CmWC-2 also interacted with CmWC-1 to form the WCC complex and was inhibited by CmVVD. In addition, CmWC-3 directly bound with the *CmPKS* promoter, but CmWC1 did not. These results suggest that albinism and fruiting body development are two independent processes; the WCC complex of CmWC-1 with CmWC-3 regulates *CmPKS* expression to regulate color change, whereas CmWC-1 with CmWC-2 affects fruiting body development *via* the carotenoid pathway. These findings will help us to better understand the albinism mechanism of *C. militaris*.

## Introduction

Light affects the growth and development of plants and most microorganisms as an energy donor and regulates fundamental processes as a light signal. During light signal transduction, photoreceptor proteins regulate vital movement by absorbing the light of particular wavelengths such as phytochromes for red/far-red light (Quail, 1991) and cryptochromes for UV and blue light (Short, 1994). After absorbing light, the conformation of the photoreceptor protein is changed, and intracellular signal transduction is induced to regulate biochemical reactions (Pudasaini and Zoltowski, 2013). Although photochemical reactions involving photoreceptor proteins are well understood, the downstream networks of the photoresponse of plants and microorganisms are quite different. For example, in *Cantharellus cibarius*, the fruiting body containing carotenoids is gold, on the contrary, it is white (Thorn et al., 2021). Similarly, carotenoids also affect the color of *Neurospora crassa* (Nelson et al., 1989). β-carotene is lacking from the fruiting body but exists in the mycelia of a white mutant *Hypsizigus marmoreus* (Lee et al., 2008). In *Aspergillus fumigates*, the conidia of the melanin biosynthesis mutant are reddish-pink, while those of the wild-type are bluish-green (Tsai et al., 1998).

Microorganisms include bacteria, viruses, fungi, some small protozoa, and microalgae. Fungi are eukaryotic organisms with eukaryotes, and no chloroplast, that sporulation, and include molds, yeasts, and mushrooms. In nature, due to the influence of environmental factors and the reproductive characteristics of fungi, albinism is common in fungi. Many studies indicate that the interaction of the photoreceptor protein white collar-1 (WC-1) with white collar-2 (WC-2) forms a white collar complex (WCC), which plays an important role in blue-light signal transduction (Talora et al., 1999). Mutations of *WC-1* and *WC-2* caused cells to be insensitive to light signals (Perkins et al., 1982; Nelson et al., 1989). WC-1 proteins can be activated by phosphorylation under light (Talora et al., 1999), and subsequently WCC regulates the expression of light-responsive genes (Chen et al., 2009; Wu et al., 2014). However, WC-1 proteins are hyperphosphorylated and subsequently become degraded under light (Talora et al., 1999). A photoreceptor protein Vivid (*VVD*) was transcribed by WCC under a light signal and feedback repressed WCC activity through the interaction with WCC (Dasgupta et al., 2015). The *VVD* mutation caused growth reduction and increased mycelium pigmentation in *Podospora anserina* (Shen et al., 2022). Nevertheless, some fungi do not possess *WC-1*/*WC-2* and/or *VVD* genes. *Candida albicans*, *Pichia stipitis*, and *Schizosaccharomyces pombe* possess none of these three genes, whereas *Coprinopsis cinerea*, *Laccaria bicolor*, *Pleurotus ostreatus*, *Cryptococcus neoformans*, *Aspergillus nidulans*, and *Chaetomium globosum* have *WC-1*/*WC-2* but not *VVD* genes (Rodriguez-Romero et al., 2010). Therefore, the light-responsive mechanism shows some variation in fungi.

Studies indicate that melanin is involved in fungal albinism. In *Colletotrichum lagenarium*, the melanin biosynthesis gene polyketide synthase (*PKS*) can recover the melanization of an albino mutant (Takano et al., 1995, 1997). In fungi, melanin is synthesized through the L-3,4-dihydroxyphenylalanine (L-dopa) or 1,8-dihydroxynaphthalene (DHN) pathways (Eisenman and Casadevall, 2012). For the L-dopa pathway, L-dopa/tyrosine is catalyzed and synthesized into dopaquinone by laccase/tyrosinase, respectively. Subsequently, dopaquinone forms melanin by oxidation and polymerization (Land et al., 2004). For the DHN pathway, acetyl CoA or malonyl CoA are first catalyzed and form 1, 3, 6, 8-tetrahydroxynaphthalene (1, 3, 6, 8-THN) through PKS. 1, 3, 6, 8-THN is then successively converted to scytalone, 1, 3, 8-THN, vermelone, and DHN through as oxidation–reduction series. Finally, melanin is produced by oxidized and polymerized DHN using phenoloxidase (Wheeler and Bell, 1988; Lundqvist et al., 1993). However, the chemistry and ultrastructure of melanin from DHN do not resemble those from L-dopa (Wheeler and Stipanovic, 1979). Therefore, melanin from the DHN and L-dopa pathways could play different roles in fungi.

Edible fungi are large fungi that can be used for food or medicine and have large fleshy or colloidal fruiting bodies or sclerotinic tissues (Zhang et al., 2021). *Cordyceps militaris*, classified into the *Cordyceps* genus of the Cordycipitaceae family, has many bioactive substances beneficial to the human body such as cordyceps acid, cordycepin, cordyceps polysaccharide, and steroids (Xiao and Zhong, 2007; Paterson, 2008; Zhou et al., 2009). Studies have indicated that the *CmWC-1* mutation caused albinism and disordered fruit body development in C. *militaris* (Yang and Dong, 2014; Yang et al., 2016). The CmWC-1 protein interacted with the AAATCAGACCAC/GTGGTCTGATTT site of its target gene promoter to regulate target gene expression in *C. militaris*, which differs from that in Neurospora crassa (Zhang et al., 2022). In this study, an albino mutant obtained by ^60^Co-γ-ray irradiation from *C. militaris* was found, and it was able to form a fruiting body. This suggested that this albino mutant may be different from the *CmWC-1* mutant previously reported. Thus, genome resequencing and transcriptomic sequencing technologies were used to search mutated genes and preliminarily analyze the gene network related to albinism in *C. militaris*. This will help us further elucidate the light-responsive mechanism of *C. militaris*.

## Materials and methods

### Materials

The strains of wild-type *C. militaris* (WT) and an albino mutant (named *Alb*) obtained by ^60^Co-γ-ray irradiation from the WT were preserved from the Edible Fungus Institute of Liaoning Academy of Agricultural Sciences. The mycelia of the WT and *Alb* strains were cultured on potato dextrose agar (PDA) medium at 22°C.

### Sample treatment

The mycelia of the WT and *Alb* strains were cultured at 22°C for 5 days under dark and then placed under light at 500–800 Lux (light 10 h/dark 14 h) at 22°C. After 2 days under light, the color of the mycelia was observed. After 10 days under light, the mycelia of the WT and *Alb* strains were collected, dried, and then weighed. Approximately 200 mg of fresh mycelia was collected before light treatment and after light for 12 h to analyze the contents of cordycepin and adenosine and were subjected to transcriptomic analysis and quantitative real-time PCR (qRT-PCR). In addition, 200 mg of fresh mycelia before light treatment was gathered and used in genome resequencing analysis. To observe the growth changes of the fruiting bodies, the WT and *Alb* liquid strains were cultured into wheat grain medium under light at 500–800 Lux (light 10 h/dark 14 h) at 22°C for 38 days. Subsequently, the height and dry weight of the fruiting bodies were analyzed. Moreover, 200 mg of fresh fruiting bodies was used in the transcriptomic and qRT-PCR analysis. All experiments had three biological replicates.

### DNA and RNA extraction

To search the mutation genes related to albinism, genomic DNA from 200 mg of the fresh mycelia of the WT and *Alb* before light treatment was extracted using the Fungi Genomic DNA Extraction Kit (Real-Times (Beijing) Biotechnology Co., Ltd., China) for genome resequencing analysis. Total RNA from the mycelia of the WT and *Alb* before and after light treatment was extracted using Trizol reagent for transcriptomic and qRT-PCR analysis. In addition, the fruiting bodies of the WT and *Alb* were utilized to extract total RNA for qRT-PCR analysis.

### Genome resequencing analysis

The sequencing library was prepared using the standard library building process of the TruSeq DNA PCR Free Prep Kit reagent (Illumina, San Diego, CA, USA). The library was first inspected using the Agilent High Sensitivity DNA Kit on an Agilent Bioanalyzer, quantified on a Promega QuantiFluor fluorescence quantitative system using the Quant-iT PicoGreen dsDNA Assay Kit to ensure a library concentration above 2 nM, and then paired-end sequenced after denaturing into a single strand by NaOH. The original offline data were preliminarily evaluated and filtered according to the sequence quality to obtain high-quality data. The high-quality data were compared with the reference genome of *C. militaris* (term = Cordyceps + militaris).^1^ Mutation sites were identified and compared, and mutation information was collected. Finally, the variation information was noted, and the corresponding annotation results were placed into a chart. GATK and ANNOVAR software were, respectively, used to detect single nucleotide polymorphisms (SNPs) and annotate the insertion and deletion (InDel) sites. In addition, the SNP and InDel sites were compared between the WT and *Alb* to eliminate any background influence. Three biological replicates were used for the WT and *Alb* samples.

### Transcriptomic sequencing analysis

Total RNA (5 μg) was utilized to build the sequencing library. The mRNA was enriched by Oligo (dT) magnetic beads and broken into approximately 300-bp fragments by ionic interruption. The first strand of cDNA was synthesized using 6-base random primer and reverse transcriptase, which served as a template to synthesize the second strand of cDNA. After the library was constructed, PCR amplification was used to enrich the library fragments, and then a 300–450 bp fragment library size was selected and analyzed for later paired-end sequencing on the Illumina HiSeq platform by Next-Generation Sequencing.

HISAT software was used to compare the sequencing data to the reference genome (see text footnote 1 term = Cordyceps + militaris), and the comparison result (Alignments) was used to assemble the transcript. To render gene expression levels between different genes and different samples comparable, fragments per kilo bases per million fragments (FPKM) was used to standardize the expression amount. The edgeR R package was used for differential expression analysis. The criteria for a differentially expressed genes were | log_2_foldchange| ≥ 1 and *P*-value < 0.05. Gene ontology (GO) and Kyoto Encyclopedia of Genes and Genomes (KEGG) enrichment analysis of differentially expressed genes were performed using KOBAS software with a corrected *P*-value < 0.05.

### 3D structure analysis of CmPKS protein

To determine whether the 677th amino acid mutation of CmPKS affects protein 3D structure, the amino acid sequences of the CmPKS normal and mutated protein were imputed the SWISS-MODEL online software^2^ and run the Start Modeling key to built PDB model. The model template alignment was 7cpx.1.A (lovastatin non-aketide synthase, polyketide synthase component). The PDB files were downloaded and opened in the Swiss-PdbViewer 4.1.0 software to built protein 3D model.

### Determination of cordycepin and adenosine contents

Dry mycelia and fruiting bodies (0.5 g) of the WT and *Alb* were ground into powder, and each was mixed with approximately 80 mL ultrapure water. The mixed solutions were placed into an ultrasonic cleaner, ultrasonically extracted for 3 h, and then combined with ultrapure water to a total volume of 100 mL. One milliliter of extract was centrifuged, and its supernatant was filtered through a 0.45 μm Millipore filter. The contents of cordycepin and adenosine were analyzed using high-performance liquid chromatography (HPLC). A C_18_ chromatographic column (250 mm × 4.6 mm, 5 μm) was used in the HPLC assay. The mobile phase was a mixture of acetonitrile and ddH_2_O (5:95, v/v), the current velocity and column temperature were, respectively, held at 1.0 mL⋅min^–1^ and 35°C, the detection wavelength was set to 260 nm, and the injection volume was 10 μL. Standard curves were made using different concentrations of standard substances of cordycepin and adenosine. The contents of cordycepin and adenosine were computed using the peak area and standard curves.

### Measurement of the carotene content

Fifty milligrams of fruiting bodies of the WT and *Alb* was used to measure carotene contents. The fruiting bodies were ground into a powder in liquid nitrogen, to which an appropriate amount of internal standard was added. The solution was extracted with 0.01% BHT (g/mL) n-hexane/acetone/ethanol mixed solution (1:1:2, v/v/v) and vortexed at room temperature for 20 min, following which the extraction was repeated once and the solution centrifuged, and then the supernatants were combined. The concentrated extract was then dissolved again in a mixture of methanol/methyl tert-butyl ether (3:1, v/v), filtered by a filter membrane (0.22 μm), and stored in a brown sample bottle for liquid chromatography-tandem mass spectrometry (LC-MS/MS) analysis.

The main conditions for liquid chromatography included column chromatography: YMC C30 (3 μm, 100 mm × 2.0 mm i.d.); mobile phase: phase A, methanol/acetonitrile (1:3, V/V) combined with 0.01% BHT and 0.1% formic acid; phase B, methyl tert-butyl ether combined with 0.01% BHT; gradient elution procedure: 0 min A/B was at 100:0 (V/V), 3 min was at 100:0 (V/V), 5 min at 30:70 (V/V), 9 min at 5:95 (V/V), 10 min at 100:0 (V/V), and 11 min at 100:0 (V/V); flow rate: 0.8 mL min^–1^; column temperature: 28°C; and injection volume: 2 mL.

The mass spectrum conditions mainly included atmospheric pressure chemical ionization (APCI) temperature 350°C and curtain gas (CUR) 25 psi. In q-trap 6,500 +, each ion pair was scanned and inspected according to optimized de-cluster voltage (declustering potential and collision energy).

### Cloning and sequencing identification of the mutated genes in *Alb*

To identify candidate genes related to the albino mutation, PCR amplification primers (Supplementary Table 1) were designed on both sides of the mutation sites and synthesized to amplify the sequences of the mutation sites. The PCR products were cloned into pMD18T vector and sequenced. DNAMMAN 8 and Chromas 2.65 software were used to compare the differences in the mutation sites between the WT and *Alb*.

### Protein pull-down assay

To determine the relationships among CmWC-1, CmWC-2, CmWC-3, and CmVVD, the CDS fragments of *CmWC-1* and *CmVVD* were inserted into the pCOLD I (His-tag) vector, and those of *CmWC-2* and *CmWC-3* were cloned into the pGEX-6P-1 (GST-tag) vector (Supplementary Table 1). Recombinant plasmids were transformed into *Escherichia coli* competent cell BL21 (DE3). Positive clone cells were cultivated into 25 mL lysogeny broth (LB) liquid medium containing 50 μg⋅mL^–1^ ampicillin at 37°C for 200 g for 12 h, and then continuously cultivated at 37°C or 16°C for 200 g for 12 h after adding 25 μL 1 M isopropyl-beta-D-thiogalactopyranoside (IPTG). The *E. coli* cells were collected and disrupted in 10 mL phosphate-buffered saline (PBS) containing 1 mg⋅mL^–1^ lysozyme using an ultrasonic crusher in an ice bath for 30 min. The supernatant was separated at 4°C for 10,000 rpm for 10 min, and then fusion proteins were purified using Ni-NTA His- or GST-Bind Resin (Merck) and recovered using 1 × PBS buffer. The extracted fusion proteins were detected by SDS-PAGE electrophoresis.

In the protein pull-down assays, 15 μL His-megbeads were washed twice using 2 mL binding buffer (50 mM Tris, 300 mM NaCl, 10 mM imidazole) to eliminate the ethanol. The His-megbeads were suspended using 100 μL 1 × PBS buffer. In the binding assay of CmWC-2 or CmWC-3 with CmWC-1 or CmVVD, 2 mL CmWC-1-His or CmVVD-His, 2 mL CmWC-2-GST or CmWC-3-GST fusion proteins, and His-megbeads were mixed together in a 15 mL centrifuge tube and shaken slightly at 4°C for 1 h. This mixture was transferred into a 2 mL centrifuge tube, the His-megbeads were adsorbed using a magnet, and then the supernatant was removed using a pipette. The His-megbeads were washed twice using 2 mL washing buffer (50 mM Tris, 300 mM NaCl, 20 mM imidazole), added to 100 μL elution buffer (50 mM Tris, 300 mM NaCl, 250 mM imidazole), shaken slightly at 4°C for 12 h, and the eluent transferred into a new 1.5 mL centrifuge tube. The proteins in the residues of the His-megbeads were separated by boiling at 100°C and then detected by western blot assay. In the control, the GST protein was used instead of the CmWC-2-GST and CmWC-3-GST fusion proteins.

### Electrophoretic mobility shift assay (EMSA)

The CmWC1-His and CmWC3-GST proteins from the protein pull-down assays were used to analyze the interaction relationship of CmWC1 and CmWC3 with the GATA box of *CmPKS* promoter. The biotin-probes, cold probes, and mutated probes of the P1 and P2 sites were synthetized (probe sequences in the Supplementary Table 1). The specificity of binding was examined by competition with the cold probes (50×) and mutated probes. EMSA was carried out using the LightShift™ EMSA Kit (Cat. #20148, Thermo Fisher, Waltham, MA, USA).

### Quantitative real-time PCR assay

Total RNA (1 μg) was used to synthesize the first strand of cDNA using the PrimeScript™ 1st Strand cDNA Synthesis Kit (TAKARA, Japan). The cDNA was used as a template in the qRT-PCR with the gene-specific primers shown in Supplementary Table 1. The qRT-PCR reactions were performed in triplicate for each sample using TB Green^®^ Premix Ex Taq™ II (Tli RNaseH Plus) (TAKARA, Japan) on an ABI 7500 system (Applied Biosystems, Waltham, MA, USA). The reaction conditions were as follows: 95°C for 30 s, 40 cycles of 95°C for 3 s and 60°C for 30 s, and 1 cycle of 60°C for 1 min. The dissociation curve was used to validate the specificity of each primer pair. Each treatment was repeated thrice. The *CmActin* gene was used as an internal control. Gene relative expression levels and significant differences between samples were computed and compared (Nolan et al., 2006).

### Bioinformatics and statistical analysis

Genome resequencing and transcriptomic data were analyzed using R.^3^ Heatmaps were produced using Pvclust package (Suzuki and Shimodaira, 2006) and pcaMethods bioconductor package (Stacklies et al., 2007). All data in this study were reported as the mean ± standard error of three biological repeats. ANOVA was used to analyze the statistically significant differences at *P* < 0.05 for the mean of each sample.

## Results

### Albino mutation (*Alb*) affects photosensitivity and fruiting body growth in *C. Militaris*

**FIGURE 1:**
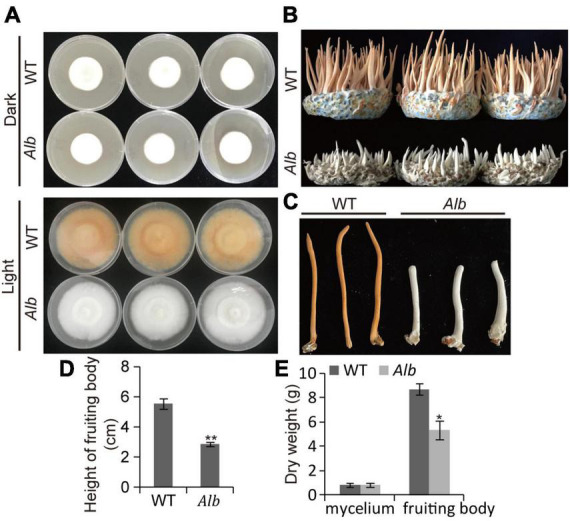
Phenotype analysis of *Alb* mutant. **(A)** Color change of the mycelia in WT and *Alb* under dark and light treatment; **(B)** fruiting body growth of WT and *Alb*; **(C,D)** height of fruiting bodies in WT and *Alb*; **(E)** dry weight of the mycelia and fruiting bodies in WT and *Alb*. WT, wild-type *C. militaris*; *Alb*, albino mutant. One and two asterisks denote significance relative to WT (Student’s *t*-test; *p < 0.05 and **p < 0.01).

An albino mutant (*Alb*) obtained by ^60^Co-γ-ray irradiation was found in *C. militaris*. Under dark conditions, the mycelia of the WT and *Alb* were white. However, the mycelia of the WT turned orange, whereas the mycelia of *Alb* remained white upon light treatment (Figure 1A). This suggested that *Alb* did not respond to the light signal. During fruiting body growth, the growth rate of the fruiting body in the WT was higher than in *Alb* (Figure 1B). In addition, the height of the fruiting body in *Alb* was significantly lower than in WT (Figures 1C, D). The dry weight of the mycelia did not differ between the WT and *Alb*, while that of the fruiting bodies was significantly reduced in *Alb* (Figure 1E). This indicated that the albino mutant repressed fruiting body growth and decreased fruiting body biomass.

### Analysis of SNP mutation sites in WT and *Alb*

**FIGURE 2:**
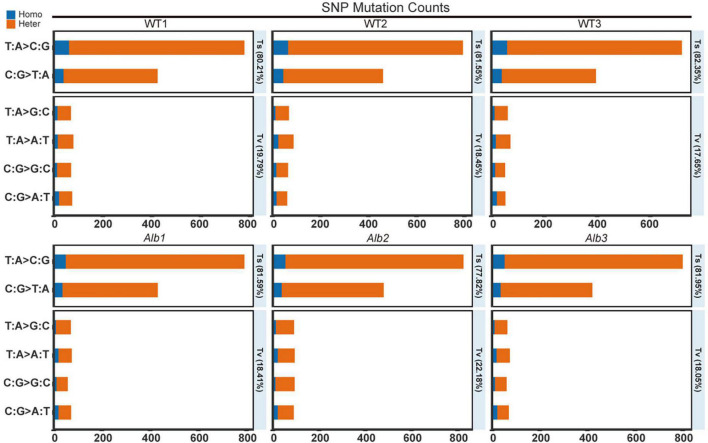
SNP mutation counts analysis between WT and *Alb*. Homo, homozygous SNP sites; Heter, heterozygous SNP sites; WT1–WT3 and Alb1–Alb3, indicate three biological repeats.

To investigate the causes of the albino phenomenon in *C. militaris*, genome resequencing technology was used here. In the SNP analysis, 1,496, 1,539, and 1,354 SNP sites were found in the three WT samples compared with the reference genome. Among these, the heterozygous SNP site counts were 1,326, 1,359, and 1,190, and the other sites constituted homozygous SNP sites in the three WT samples (Supplementary Table 2). Among these sites, transitions (Ts) site counts were 1,200, 1,255, and 1,115, and transversions (Tv) site counts were 296, 284, and 239 in the three WT samples (Supplementary Table 2). The frequency of the Ts mutations reached 80.21, 81.55, and 82.35%, while that of the Tv mutations was only 19.79, 18.45, and 17.65% in the three WT samples (Figure 2). These results suggested that the wild-type *C. militaris* exhibited a lot of natural SNP variation.

In the three *Alb* mutants, 1,499, 1,677, and 1,489 SNP sites were found compared with the reference genome. The heterozygous SNP site counts were 1,353, 1,517, and 1,338, and the homozygous SNP sites were 146, 160, and 151 (Supplementary Table 2). In addition, the Ts sites were 1,223, 1,305, and 1,221, and the Tv sites were 276, 372, and 269 (Supplementary Table 2). The Ts frequency was 81.59, 77.82, and 81.95%, and the Tv frequency was 18.41, 22.18, and 18.05% (Figure 2). These results suggested that *Alb* remained present in many SNP variations, and most of the variation may be similar to that of the WT.

Among all SNP mutation sites, 190, 347, 1,768, 159, 206, and 40 sites were, respectively, located in the gene downstream region, exon, intergenic region, intron, upstream region, and between the upstream region and its neighboring gene downstream region (Supplementary Table 3). In addition, only two sites belonged to splicing sites (Supplementary Table 3).

### Analysis of InDel mutation sites in WT and *Alb*

During the genome resequencing analysis, many InDel sites were found in the WT and *Alb*: there were 3,659, 3,679, and 3,662 InDel sites in the three WT samples and 3,662, 3,647, and 3,664 InDel sites in the three *Alb* samples. Among these sites, 3,121, 3,132, and 3,141 sites in WT and 3,149, 3,090, and 3,146 sites in *Alb* were homozygous sites, while the remainder were heterozygous sites. A total of 2,833, 2,871, and 2,855 sites in WT and 2,849, 2,832, and 2,849 sites in *Alb* were insertion mutations, while the other sites were deletion mutations (Supplementary Table 4).

Among these InDel sites, 610, 99, 867, 494, 633, and 299 sites were, respectively, located in the downstream region, exon, intergenic region, intron, upstream region, and between the upstream region and its neighboring downstream gene (Supplementary Table 5). In addition, 903 sites were not located inside genes and between two genes, and seven sites belonged to splicing sites.

### Identification of mutant genes in *Alb*

To screen mutation genes related to the albino phenomenon, all SNP and InDel sites were compared between the WT and *Alb* to overcome interference of WT natural variation. By comparison, exon mutations in four genes were only detected in *Alb*, while those of the others between WT and *Alb* did not differ or were the same as the reference genome.

**FIGURE 3:**
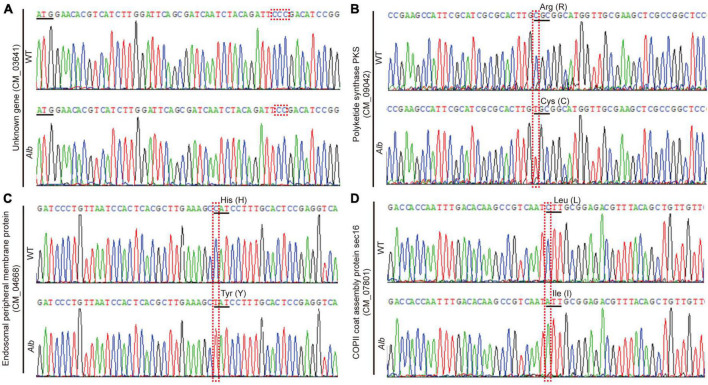
Sequencing analysis of four mutation genes in WT and *Alb*. **(A–D)** Unknown gene (CM_03641), polyketide synthase (CCM_09042), endosomal peripheral membrane protein (CCM_04668), and COPII coat assembly protein sec16 (CCM_07801). WT, wild-type *C. militaris*; *Alb*, albino mutant.

Among the four mutation genes, an unknown gene (CM_03641) belonged to an InDel mutation and missed one base (C) distance from ATG at 43 bp, which caused a frameshift mutation (Figure 3A). The other three genes belonged to the SNP mutation, and these SNP site mutations only caused the amino acid change. Among the three mutation genes, the full-length sequences of polyketide synthase (CCM_09042), endosomal peripheral membrane protein (CCM_04668), and COPII coat assembly protein sec16 (CCM_07801) were 7,464, 5,148, and 6,909 bp, respectively. In *Alb*, the 2,029th nucleotide of polyketide synthase changed from C to T, leading to an amino acid change from original Arg (R) to Cys (C) (Figure 3B). R is an alkaline amino acid and its isoelectric point (PI) is 10.76, while C is a sulfur-containing amino acid and its PI is 5.02. Thus, this change would affect the space conformation of polyketide synthase. Similarly, nucleotide 1,582th of the endosomal peripheral membrane protein changed to polyketide synthase, but the amino acid corresponding to this site changed from His (H) to Tyr (Y) (Figure 3C). H is also an alkaline amino acid and its PI is 7.59, while Y is an aromatic amino acid and its PI is 5.66. This change may affect protein space conformation. In addition, although the 433rd nucleotide of the COPII coat assembly protein sec16 changed from C to A, and the amino acid corresponding to this site changed from Leu (L) to Ile (I) (Figure 3D), and L and I had similar characteristics. Thus, this mutation could not affect protein space conformation.

### The albino mutation affected the expression level of many genes

To understand which regulatory networks were altered in *Alb*, RNA-Seq was used. The FPKM values of 9,651 genes were analyzed in the mycelia of the WT and *Alb* before and after light treatment (Supplementary Table 6). Before light treatment, 9,349 and 9,319 genes were expressed in the WT and *Alb* samples, respectively. After light treatment, 9,408 and 9,430 genes were not detected in the three WT and Alb samples, respectively (Supplementary Table 6). These results suggested that the expression of some genes in *C. militaris* may be induced by light signals.

**FIGURE 4:**
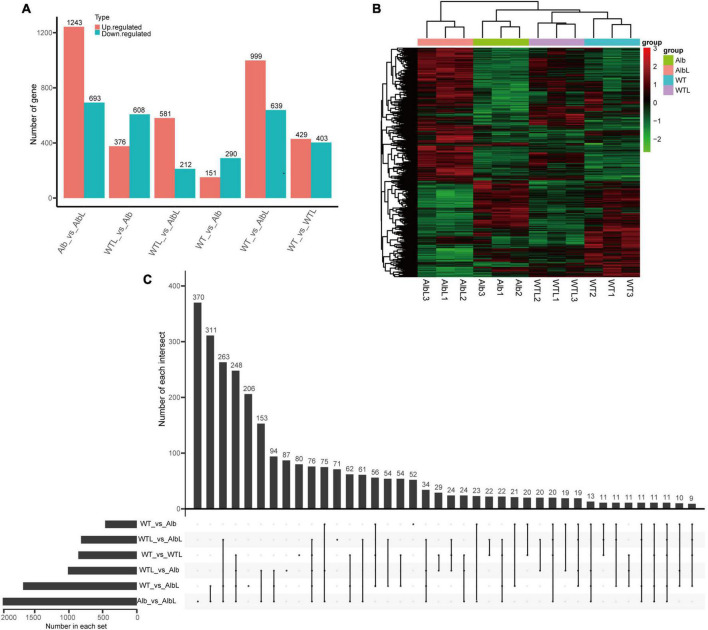
Analysis of differential expression genes in WT and *Alb* before and after light treatment. **(A)** Statistics of the number of differentially expressed genes among different groups; **(B)** cluster analysis of differentially expressed genes in all samples; **(C)** analysis of differentially expressed gene number among multiple group. Number of each intersection, indicates the number of common differential genes identified by multiple comparison groups. Number in each set, indicates number of all differential genes identified in each group. WT, the mycelia of wild-type *C. militaris* before light treatment; WTL, the mycelia of wild-type *C. militaris* after light treatment; *Alb*, the mycelia of the albino mutant before light treatment; AlbL, the mycelia of the albino mutant after light treatment.

Before light treatment, 151 up-regulated and 290 down-regulated genes were found in the Alb sample compared with the WT (Figure 4A and Supplementary Table 7). These genes were mainly enriched in oxidoreductase activity, oxidation-reduction process, membrane, intrinsic and integral component of membrane, and catalytic activity in the GO analysis (Supplementary Figure 1A), and lysine biosynthesis, tryptophan metabolism, and phenylalanine, tyrosine and tryptophan biosynthesis in the KEGG analysis (Supplementary Figure 2A). This indicated that the change in expression level of these genes may affect the growth and development of the mycelia in *Alb*. After light treatment, 429 up-regulated and 403 down-regulated genes were found in the WTL sample (Figure 4A and Supplementary Table 8) and were mainly involved in membrane, intrinsic and integral component of membrane, catalytic activity (Supplementary Figure 1B), and glycine, serine, and threonine metabolism pathway (Supplementary Figure 2B). However, 999 up-regulated and 639 down-regulated genes were found in the AlbL sample compared with the WT before light treatment (Figure 4A and Supplementary Table 9) and were enriched in membrane, intrinsic and integral component of membrane, catalytic activity (Supplementary Figure 1C), and peroxisome, glycine, serine, threonine, tryptophan, tyrosine, phenylalanine, cyanoamino acid, nitrogen, amino sugar, and nucleotide sugar metabolism pathways (Supplementary Figure 2C). Interestingly, 1,243 up-regulated and 693 down-regulated genes were found in the AlbL sample compared with Alb before light treatment (Figure 4A and Supplementary Table 10) and were mainly enriched in membrane, intrinsic and integral component of membrane, catalytic activity (Supplementary Figure 1D), glycerolipid, pyruvate, glyoxylate, dicarboxylate, glycolysis, gluconeogenesis, phenylalanine, glycine, serine, threonine, tyrosine, amino sugar, nucleotide sugar, arginine, proline, pantothenate and CoA biosynthesis, and tryptophan metabolism pathways (Supplementary Figure 2D). This suggested that the expression of many genes in the mycelia was induced under light, while the expression levels of more genes were changed. However, these changes did not induce mycelium color change in *C. militaris*. In addition, 376 up-regulated and 608 down-regulated genes were found in the Alb sample compared with the after-light WTL sample (Figure 4A and Supplementary Table 11) and were mostly involved in membrane, intrinsic and integral component of membrane, catalytic activity (Supplementary Figure 1E), arginine, proline, glyoxylate, dicarboxylate, peroxisome, tryptophan, phenylalanine, tyrosine, cysteine, methionine, glycine, serine, threonine metabolism, pantothenate, CoA, valine, leucine, and isoleucine biosynthesis, and protein processing in endoplasmic reticulum pathways (Supplementary Figure 2E). A total of 581 up-regulated and 212 down-regulated genes were found in the AlbL sample compared with the after-light WTL sample (Figure 4A and Supplementary Table 12) and were mainly involved in oxidoreductase activity, membrane, intrinsic and integral component of membrane (Supplementary Figure 1F), and amino sugar and nucleotide sugar metabolism (Supplementary Figure 2F).

To compare the relationships between these differentially expressed genes in different samples, the differential genes of all samples were analyzed by two-way clustering. The results indicated that the differentially expressed genes of the three biological repeats of every sample clustered together and showed similar expression patterns (Figure 4B). In addition, the number of common differentially expressed genes among the comparison groups was also analyzed. There were 52, 71, 80, 87, 206, and 370 differential genes that only showed in WT vs. Alb, WTL vs. AlbL, WT vs. WTL, WTL vs. Alb, WT vs. AlbL, and Alb vs. AlbL, respectively (Figure 4C). These results suggested that these genes may be involved in fruiting body development and the light signal pathway.

### The polyketide synthase (CmPKS) mutation affected melanin accumulation in *Alb*

**FIGURE 5:**
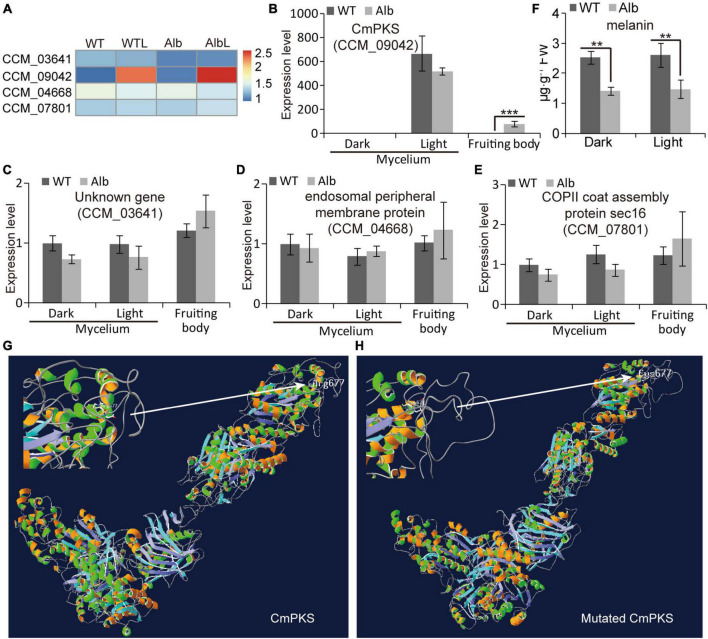
Expression analysis of four mutated genes under dark and light treatment. **(A)** Heatmap analysis of CCM_03641, CCM_09042, CCM_04668, and CCM_07801 based on their FPKM values in the WT, WTL, *Alb*, and AlbL samples; **(B–E)** expression analysis of the four genes in the mycelia under dark and light treatment, and fruiting body; **(F)** compare of melanin content between WT and *Alb* mycelia under dark and light conditions; **(G,H)** 3D model of the normal and mutated CmPKS proteins. Two asterisks denote significance relative to WT (Student’s *t*-test; **p < 0.01).

In the SNP analysis, four genes (CCM_03641, CCM_09042, CCM_04668, and CCM_07801) showed mutations in the *Alb* sample. In the RNA-Seq analysis, the FPKM values of these four genes were unchanged in the mycelia of the WT and *Alb* under dark and light conditions (Figure 5A). The *CmPKS* (CCM_09042) expression level did not differ in the mycelia of the WT and *Alb* under dark and light, while it was induced by light. Meanwhile, the expression level of *CmPKS* was significantly increased in the *Alb* fruiting body (Figure 5B). This suggested that *CmPKS* responded to light signals and was involved in the growth and development of the fruiting body. However, the expression levels of the other three genes did not differ in all samples (Figures 5C–E). This indicated that the three genes were not involved in the response to light signals and the development of the fruiting body. To determine whether the mutation of *CmPKS* affected melanin biosynthesis, the content of melanin was analyzed in the mycelia of the WT and *Alb* under dark and light conditions. Although the content of melanin in *Alb* was significantly lower than in the WT under dark and light conditions (Figure 5F), the light signal did not induce melanin biosynthesis. In addition, the 677th Arg (R) of the CmPKS protein could compose hydrogen bonds with the 673th Ile, 674th Ala, and 678th Gly (Figure 5G), while the 677th Cys (C) of the mutated CmPKS protein composed hydrogen bonds with the 672th Arg, 673th Ile, 674th Ala, 675th His, 676th Leu, 678th Gly, and 679th Met in the 3D model (Figure 5H). These results suggested that the 677th amino acid change of the CmPKS protein affected protein 3D structure and melanin or other ketone metabolites synthesized through CmPKS did regulate the color change from white to orange under light.

### Transcript of the WCC was not affected in *Alb*

**FIGURE 6:**
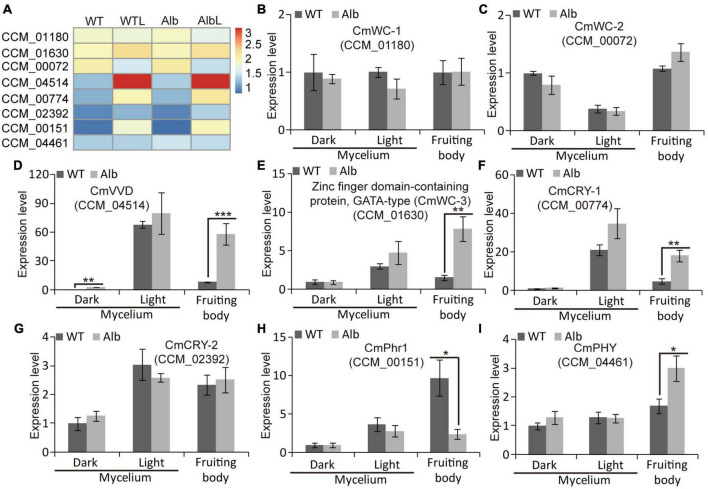
Expression analysis of photoresponsive genes in WT and *Alb*. **(A)** Heatmap analysis of *CmWC-1* (CCM_01180), *CmWC-2* (CCM_00072), *CmVVD* (CCM_04514), *CmWC-3* (CCM_01630), *CmCRY-1* (CCM_00774), *CmCRY-2* (CCM_02392), *CmPhr1* (CCM_00151), and *CmPHY* (CCM_04461) based on their FPKM values in the WT, WTL, *Alb*, and AlbL samples; **(B–I)** expression analysis of the eight genes in the mycelia under dark and light treatment, and fruiting body. One, two, and three asterisks denote significance relative to WT (Student’s *t*-test; *p < 0.05, **p < 0.01, and ***p < 0.001).

Under dark and light conditions, the expression levels of *CmWC-1* (CCM_01180) and *CmWC-2* (CCM_00072) did not differ in the mycelia of the WT and *Alb*, whereas the expression of *CmWC-2* was reduced under light (Figures 6A–C). In addition, their expression levels in the fruiting body were similar to those in the mycelia. This suggested that the light signal could inhibit *CmWC-2* transcription in the mycelia, but did not act in the fruiting body. Interestingly, the expression of *CmVVD* (CCM_04514) was significantly increased under dark conditions in the mycelia of *Alb* (Figures 6A, D). Although *CmVVD* expression showed no difference in the mycelia of WT and *Alb*, it was significantly increased under light treatment. In the fruiting body, *CmVVD* expression was higher in *Alb* than in WT (Figure 6D). These results indicated that *CmVVD* might be involved in photoreception and the developmental regulation of the fruiting body.

In the mycelia of WT and *Alb*, the expression of a zinc-finger domain-containing protein CCM_01630 (named *CmWC-3*) was induced by the light signal, while its expression did not differ between WT and *Alb* under light (Figures 6A, E). However, *CmWC-3* expression was significantly improved in the fruiting body of *Alb*. This result suggested that *CmWC-3* may also play a regulatory function similar to *CmVVD*. In addition, two cryptochrome genes CCM_00774 (*CmCRY-1*) and CCM_02392 (*CmCRY-2*) were induced by a light signal in the mycelia of the WT and *Alb* (Figures 6A, F, G), but *CmCRY-1* expression was significantly higher in the fruiting body of *Alb* than in the fruiting body of WT (Figure 6F). This suggested that *CmCRY-1* may also be involved in regulating the development of the fruiting body.

Under dark and light conditions, the expression levels of deoxyribodipyrimidine photo-lyase CCM_00151 (*CmPhr1*) and sensor histidine kinase/response regulator CCM_04461 (*CmPHY*) did not differ in the mycelia of the WT and *Alb*, whereas *CmPhr1* expression was increased by the light signal (Figures 6A, H, I). However, *CmPhr1* expression was significantly improved in the fruiting body of the WT, while *CmPHY* expression was increased in the fruiting body of Alb (Figures 6A, H, I). These results indicated that *CmPhr1* responded to light induction and was involved in regulating the development of the fruiting body. Its expression was also affected by the albino mutation, which is in contrast to *CmPHY*.

### CmWC-3 is involved in WCC formation by interaction with WC-1

**FIGURE 7:**
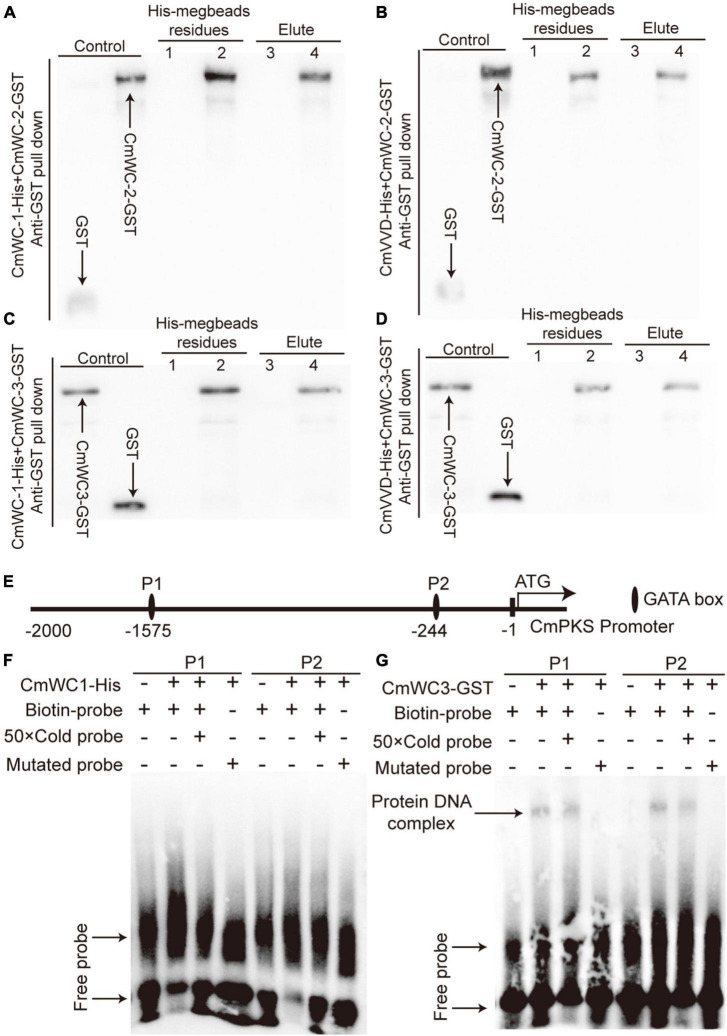
The relationship of CmWC-1, CmWC-2, CmWC-3, CmVVD, and CmPKS. Panels **(A–D)** respectively indicates the protein pull-down result of CmWC-1 with CmWC-2, CmVVD with CmWC-2, CmWC-1 with CmWC-3, and CmVVD with CmWC-3. Lane 1, GST + CmWC-1-His or CmVVD-His megbeads; lane 2, CmWC-2-GST or CmWC-3-GST + CmWC-1-His or CmVVD-His megbeads; lane 3, GST + CmWC-1-His or CmVVD-His elute; lane 4, CmWC-2-GST or CmWC-3-GST + CmWC-1-His or CmVVD-His elute. GST, CmWC-2-GST, and CmW-C3-GST as a control. **(E)** The GATA box analysis of the CmPKS promoter. **(F–G)** The binding analysis of CmWC1 and CmWC3 with two GATA box of the CmPKS promoter. P1 and P2, indicate the GATA box.

To determine the relationships among CmWC-1, CmWC-2, CmWC-3, and CmVVD, a protein pull-down assay was performed. After CmWC-1-His, CmWC-2-GST, and His-megbeads were incubated together *in vitro*, both the eluent and residues from the His-megbeads could detect CmWC-2-GST by western blot assay. However, the combined signal was not detected after replacing CmWC-2-GST with the GST-tag protein (Figure 7A). This indicates that CmWC-2 interacted with CmWC-1 in *C. militaris*. The CmWC-2-GST protein was detected in the eluent and residues from the His-megbeads when CmVVD-His instead of CmWC-1-His was used (Figure 7B). Thus, CmVVD also interacted with CmWC-2. In addition, CmWC-3 was present in the eluent and residues from the His-megbeads when CmWC-3-GST instead of CmWC-2-GST was used in the protein pull-down assay (Figures 7C, D). This suggests that CmWC-3 also interacts with CmWC-1 and CmVVD. Thus, CmWC-1 not only interacts with CmWC-2 to form the WCC complex but also interacts with CmWC-3 to form this complex. Moreover, CmVVD inhibits the activity of CmWC-2 and CmWC-3 by interacting with them.

### CmWC-3 directly bound with the *CmPKS* promoter

To determine whether the WCC complex of CmWC1 with CmWC3 regulates *CmPKS* expression, an EMSA assay was performed. There are two GATA boxes in the transcriptional regulation region of *CmPKS* promoter (Figure 7E). In EMSA assay, CmWC1-His protein did not bind with any of the two GATA boxes (Figure 7F), but CmWC3-GST protein could bind with the two GATA boxes (Figure 7G). These results indicated that the WCC complex of CmWC1 with CmWC3 regulated *CmPKS* expression by CmWC3 directly binding with the GATA box of *CmPKS* promoter.

### The albino mutation affected cordycepin accumulation in *Alb*

**FIGURE 8:**
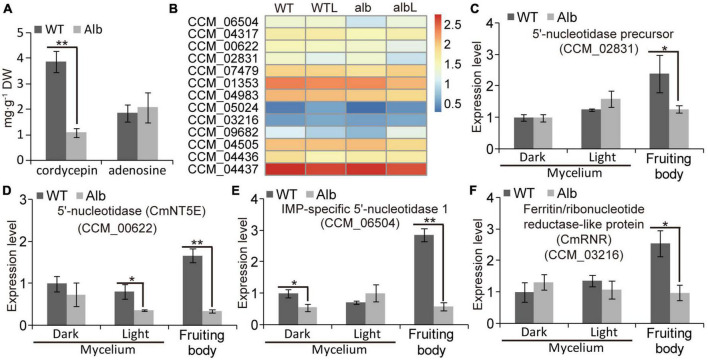
Expression analysis of genes related cordycepin biosynthesis. **(A)** Compare of cordycepin and adenosine contents in the fruiting body of WT and *Alb*; **(B)** heatmap analysis of CCM_06504, CCM_04317, *CmNT5E* (CCM_00622), CCM_02831, CCM_007479, CCM_01353, CCM_04983, CCM_05024, CCM_03216, CCM_09682, CCM_04505, CCM_04436, and CCM_04437 based on their FPKM values in the WT, WTL, *Alb*, and AlbL samples; **(C–F)** expression analysis of CCM_02831, *CmNT5E* (CCM_00622), CCM_06504, and CCM_03216 in the mycelia under dark and light treatment, and fruiting body. One and two asterisks denote significance relative to WT (Student’s *t*-test; *p < 0.05 and **p < 0.01).

To explore the effect of color change on cordycepin biosynthesis, the contents of cordycepin and adenosine were analyzed. In the fruiting body, cordycepin was significantly reduced in *Alb*, while adenosine did not differ between WT and *Alb* (Figure 8A). This indicated that the albino mutation repressed cordycepin biosynthesis.

The FPKM values of 13 genes related to cordycepin biosynthesis were compared between WT and *Alb* under dark and light conditions (Figure 8B). Under dark, only IMP-specific 5′-nucleotidase 1 (CCM_06504) and adenosine nucleosidase (CCM_09682) were down-regulated in the mycelia of *Alb*, while other genes exhibited no difference in expression between WT and *Alb* (Figure 8B). Under light, CCM_09682 was up-regulated in the mycelia of Alb (Figure 8B), whereas other genes did not differ in expression (Figure 8B). These results suggested that the expression of most genes related to cordycepin biosynthesis was not affected by the light signal and albino mutation.

A similar phenomenon was observed in the qRT-PCR assay (Figures 8C–F and Supplementary Figures 4A–I). In the fruiting body, the expression levels of 5′-nucleotidase precursor (CCM_02831), 5′-nucleotidase *CmNT5E* (CCM_00622), CCM_06504, and ferritin/ribonucleotide reductase-like protein (CCM_03216) were lower in *Alb* than in WT (Figures 8C–F), and the expression levels of other genes were not differ (Supplementary Figures 3A–I). These results indicated that the reason for the reduction in the cordycepin content in the fruiting body of *Alb* may be the low expression of CCM_02831, *CmNT5E*, CCM_06504, and CCM_03216.

### The albino mutation did not affect carotenoid biosynthesis in *Alb*

To determine the effect of the albino mutant on carotenoids, the FPKM values of 13 genes related to carotenoid biosynthesis including dioxygenase (CCM_06728), fatty aldehyde dehydrogenase (CCM_09155), geranylgeranyl pyrophosphate synthetase GGPPS (CCM_03697, CCM_03059, and CCM_06355), hydroxymethylglutaryl-CoA synthase (CCM_00310), farnesyl pyrophosphate synthetase (CCM_03203), 3-hydroxy-3-methylglutaryl-coenzyme A reductase (CCM_04994), acetyl-CoA acetyltransferase (CCM_05367 and CCM_07659), phosphomevalonate kinase (CCM_05380), mevalonate kinase (CCM_07937), and diphosphomevalonate decarboxylase (CCM_08582) were analyzed in the mycelia of the WT and *Alb* under dark and light conditions. The expression of these genes did not show any obvious difference between the WT and *Alb* (Supplementary Figure 4A). In addition, the products of carotenoid biosynthesis and metabolism were measured by LC-MS/MS analysis. However, only phytofluene was detected in the fruiting body of the WT and *Alb*, and its content did not show any difference between the WT and *Alb* (Supplementary Figure 4B). Lutein oleate, lutein decanoate, lutein stearate, violaxanthin myristate, and keratin were also detected and exhibited no difference between the WT and *Alb* (Supplementary Figure 4C). These results suggested that carotenoid biosynthesis and metabolism were not affected during the color change from white to orange, and carotenoids were not involved in regulating the color change process.

## Discussion

Some edible fungi, such as *Volvariella volvacea*, *Ganoderma lucidum*, *Lentinula edodes*, and *C. militaris* require a certain level of light to grow and develop. However, photoreaction studies have mainly focused on the model fungus *N. crassa*, whereas there have been comparatively fewer studies on edible fungi.

Studies suggest that the VVD-WCC model plays an important role in regulating fungal photoreactions. Under light, photoreceptor protein WC-1 is activated by phosphorylation and interacts with WC-2 to form the WCC complex, improving the transcription of *VVD* and other light-response genes. The VVD feedback inhibits WCC stability by interacting with WCC to promote WC-1 degradation (Dasgupta et al., 2015). In *C. militaris*, the mutation of *CmWC-1* resulted in the failure of color conversion of the mycelia, and a fruiting body was not formed under light (Yang et al., 2016), which affected the expression of most light-response genes (Wang et al., 2018; Zhang et al., 2022). Therefore, *CmWC-1* is involved in the photoregulatory network and fruiting body development. In this study, the albino mutant *Alb* from *C. militaris* showed albinism (Figure 1A), while its fruiting body could develop under light (Figures 1B, C). This suggests that *CmWC-1* may not be mutated in the *Alb* mutant. To determine the mutated genes, genome resequencing technology was used in this study. During InDel analysis, an unknown gene (CM_03641) deleted a base and caused a frameshift mutation (Figure 3A). During the SNP analysis, *CmPKS* (CCM_09042), endosomal peripheral membrane protein (CCM_04668), and COPII coat assembly protein sec16 (CCM_07801) incurred single base replacement, resulting in a change in the triplet codon at this location (Figures 3B–D). However, only the expression of *CmPKS* was induced under light among these genes (Figures 5A–E). In addition, *CmWC-1*, *CmWC-2*, and *CmVVD* were not mutated in our albino mutant *Alb*. In the transcriptomic sequencing and qRT-PCR analysis, the expressions of *CmWC-1* and *CmWC-2* did not differ between the WT and *Alb*, but light signal could repress the expression of *CmWC-2* (Figures 6A–C). In addition, *CmVVD* expression in the *Alb* mutant was significantly up-regulated under dark and in the fruiting body (Figure 6D). Thus, we conjecture that the *CmPKS* mutation may be the direct cause of albinism, and CmVVD may affect CmPKS activity *via* the VVD-WCC model. However, CmWC-2 may be replaced by CmWC-3 in the WCC model, because *CmWC*-*2* expression was inhibited (Figure 6C) and *CmWC-3* was induced by a light signal (Figure 6E). In the protein pull-down assay, we found that both CmWC-2 and CmWC-3 could interact with CmWC-1 and CmVVD (Figures 7A–D). This suggests that CmWC-3 also binds with CmWC-1 to form the WCC complex and is inhibited by CmVVD under light. In addition, CmWC-3 could bind with the *CmPKS* promoter, while CmWC-1 did not in the EMSA assay (Figures 7F, G). Thus, an increased expression of *CmWC-3* will promote the transcription of downstream genes *CmPKS* under light.

Although many studies have focused on fungal albinism, the underlying causes of albinism are always divergent. A mutation in the carotenoid biosynthetic enzyme geranylgeranyl pyrophosphate (GGPP) synthetase (*al-3*) caused albinism in *N. crassa* (Nelson et al., 1989). This suggests that carotenoids may be a key factor affecting albinism in *N. crassa*. However, the mutant of the melanin biosynthetic enzyme *PKS* also showed an albino phenotype in *Colletotrichum lagenarium* and *Aspergillus fumigatus* (Takano et al., 1995, 1997; Tsai et al., 1998). Thus, melanin plays an important role in color change under light. We found that the melanin content was reduced in the *Alb* mutant before and after light treatment (Figure 5F). Among the products of carotenoid biosynthesis and metabolism, a few metabolites such as phytofluene, lutein oleate, lutein decanoate, lutein stearate, violaxanthin myristate, and keratin could be detected between the WT and *Alb*. Moreover, their contents did not differ (Supplementary Figures 4B, C). The expression level of many genes related to carotenoid biosynthesis and metabolism also exhibited no difference between WT and *Alb* before and after light treatment (Supplementary Figure 4A). Thus, melanin from the DHN pathway may be the fundamental factor affecting the albinism of *C. militaris*, but not carotenoids.

Cordycepin, as a nucleoside antibiotic, has many pharmacological activities, such as antibacterial, anti-inflammatory, anti-virus, anti-tumor, and immune regulation (Qin et al., 2019; Tan et al., 2020). However, the biosynthetic pathway of cordycepin is still divergent. In earlier studies, cordycepin was polymerized *via* 3′-deoxyribose and adenine using a glycosidic bond (Kredich and Guarino, 1961). However, adenosine can also be used as the precursor of cordycepin synthesis and produces cordycepin through multiple enzymes such as adenylate kinase (ADEK), ferritin/ribonucleotide reductase-like protein (RNR), 5’-nucleotidase (NT5E), adenosine nucleosidase, oxidoreductase domain-containing protein (Cns1), and phosphoribosyl-aminoimidazole-succinocarboxamide synthase (Cns2) (Suhadolnik and Cory, 1964; Lennon and Suhadolnik, 1976; Kato et al., 2017; Liu et al., 2018). In addition, a mutation of *CmWC-1* repressed cordycepin biosynthesis and fruiting body development (Yang et al., 2016). In this study, the mutation of *CmPKS* reduced the cordycepin content and the height and biomass of the fruiting body, but did not affect fruiting body development (Figures 1B–E). Moreover, the expression levels of the 5’-nucleotidase precursor, *CmNT5E*, IMP-specific 5’-nucleotidase 1, and *CmRNR* were inhibited in the *Alb* mutant (Figures 8C–F), while those of the others were unchanged (Supplementary Figures 3A–I). Thus, melanin or its precursors may be involved in regulating the synthesis of cordycepin.

**FIGURE 9:**
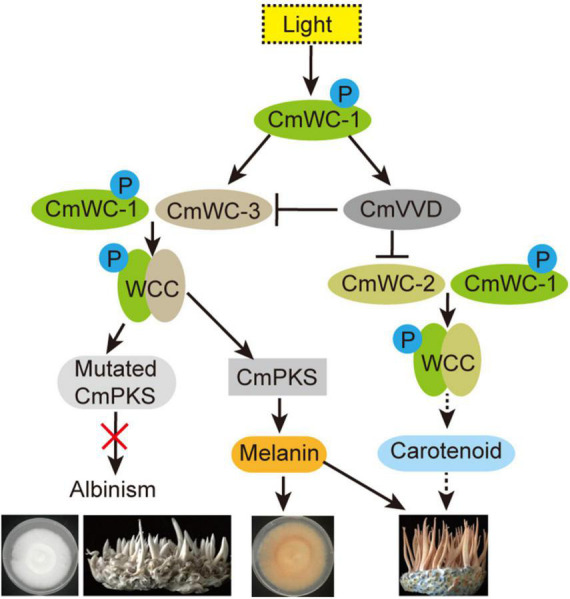
Regulation network of albinism in *C. militaris*. P, indicates phosphorylation; black one-way solid line, positive regulation; black one-way dotted line, maybe positive regulation; black line ending with bar, indicates inhibition; red fork, function loss of protein.

In summary, our results suggest that the key factor of albinism in *C. militaris* may be melanin or its precursors, but not carotenoids. Under light, CmWC-1 proteins were activated by phosphorylation, following which CmWC-1 enhanced *CmWC-3* transcription and formed a WCC complex together with CmWC-3, which positively regulated *CmPKS* transcription. Increased *CmPKS* promoted the involvement of melanin biosynthesis in the color conversion of the mycelia and fruiting body of *C. militaris* and melanin partly affected the cordycepin biosynthesis. Mutated *CmPKS* cause albinism of the mycelia and fruiting bodies under light. In addition, CmWC-1 also improved CmVVD expression, the WCC complex of CmWC-1 with CmWC-2 was inhibited by CmVVD and regulated cordycepin biosynthesis, and cordycepin affected the growth of fruiting body in *C. militaris*. In addition, CmVVD repressed the WCC complex of CmWC-1 with CmWC-3 by interacting with CmWC-3 (Figure 9). These findings will help us further elucidate the molecular mechanism of growth and development in *C. militaris*.

## Data availability statement

The original contributions presented in this study are included in the article/Supplementary material, further inquiries can be directed to the corresponding author. The accession numbers of genome resequencing and transcriptome data are PRJNA946173 and PRJNA946167, respectively.

## Author contributions

JX and YL designed the total experiments. YZ and XC planned, conducted, and analyzed the genome resequencing and transcriptomics data. YZ, XC, and YL finished the qRT-PCR, protein pull-down, EMSA, and LC-MS/MS assays. JX, YZ, and XC finished the HPLC and melanin treatment assays. YZ wrote the manuscript. All authors contributed to the article and approved the submitted version.

## Footnotes

1
https://www.ncbi.nlm.nih.gov/genome/?term=Cordyceps+militaris

2
https://www.swissmodel.expasy.org

3
http://www.r-project.org/

## Supplementary material

Supplementary Figure 1 The GO enrichment analysis of the WT and *Alb* samples before and after light treatment. **(A–F)** Respectively indicates the GO enrichment in WT vs. *Alb*, WT vs. WTL, WT vs. AlbL, *Alb* vs. AlbL. WTL vs. *Alb*, and WTL vs. AlbL. WT, the mycelia of wild-type *C. militaris* before light; WTL, the mycelia of wild-type *C. militaris* after light; *Alb*, the mycelia of albino mutant before light; AlbL, the mycelia of albino mutant after light.

Supplementary Figure 2 The KEGG pathway enrichment analysis of the WT and *Alb* samples before and after light treatment. **(A–F)** Respectively indicates the KEGG pathway enrichment in WT vs. *Alb*, WT vs. WTL, WT vs. AlbL, *Alb* vs. AlbL. WTL vs. *Alb*, and WTL vs. AlbL.

Supplementary Figure 3 Expression analysis of cordycepin biosynthesis genes in WT and *Alb*. **(A–I)** Respectively indicates the expression levels of pyrimidine 5′-nucleotidase (CCM_04317), ferritin/ribonucleotide reductase-like protein (CCM_05024), Adenosine nucleosidase (CCM_09682), adenylate kinase (CCM_07479, CCM_01353, and CCM_04983), purine nucleoside phosphorylase (CCM_04505), oxidoreductase domain- containing protein (Cns1) (CCM_04436), and phosphoribosyl-aminoimidazole-succinocarboxamide synthase (Cns2) (CCM_04437). One asterisks denote significance relative to WT (Student’s *t*-test; **p* < 0.05).

Supplementary Figure 4 Analysis of the products and related gene expression in carotenoid biosynthesis and metabolism. **(A)** Heatmap analysis of dioxygenase (CCM_06728), fatty aldehyde dehydrogenase (CCM_09155), GGPP synthetase (CCM_03697, CCM_03059, and CCM_06355), hydroxymethylglutaryl-CoA synthase (CCM_00310), FPP synthetase (CCM_03203), 3-hydroxy-3-methylglutaryl-coenzyme A reductase (CCM_04994), acetyl-CoA acetyltransferase (CCM_05367), phosphomevalonate kinase (CCM_05380), acetyl-CoA acetyltransferase (CCM_07659), mevalonate kinase (CCM_07937), and diphosphomevalonate decarboxylase (CCM_08582) in the mycelia and fruiting body of WT and *Alb* before and after light treatment. **(B,C)** Respectively indicated the product contents of carotenoid biosynthesis and metabolism.
