# Supplementary figures and images for: Genome resequencing and transcriptome analysis reveal the molecular mechanism of albinism in Cordyceps militaris
Source: Front Microbiol. 2023 Apr 11;14:1153153. doi: 10.3389/fmicb.2023.1153153 (PMC10126257; doi:10.3389/fmicb.2023.1153153)

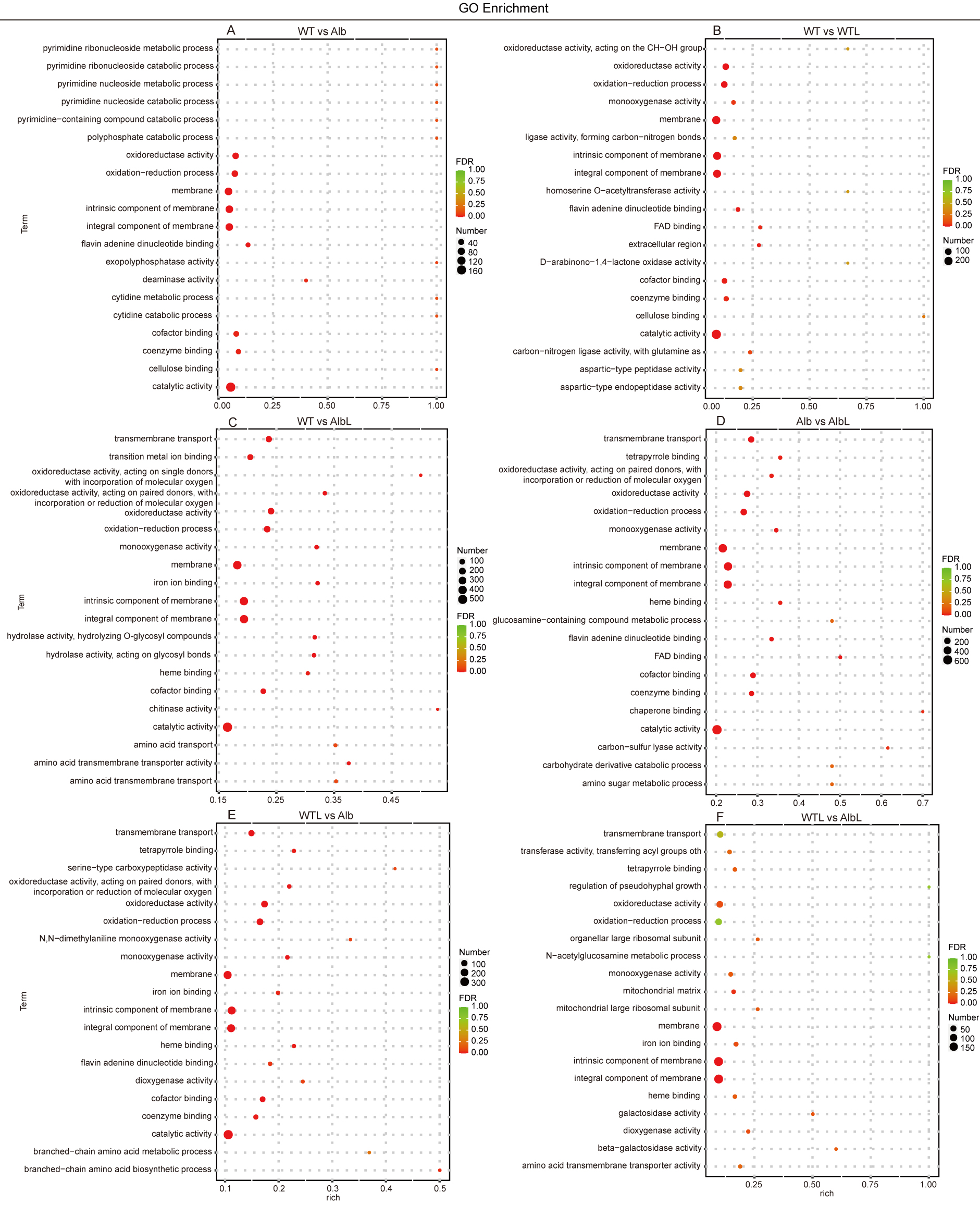

Supplement: Supplementary Figure 1 — The GO enrichment analysis of the WT and Alb samples before and after light treatment. (A–F) Respectively indicates the GO enrichment in WT vs. Alb, WT vs. WTL, WT vs. AlbL, Alb vs. AlbL. WTL vs. Alb, and WTL vs. AlbL. WT, the mycelia of wild-type C. militaris before light; WTL, the mycelia of wild-type C. militaris after light; Alb, the mycelia of albino mutant before light; AlbL, the mycelia of albino mutant after light. [file Image_1.JPEG]

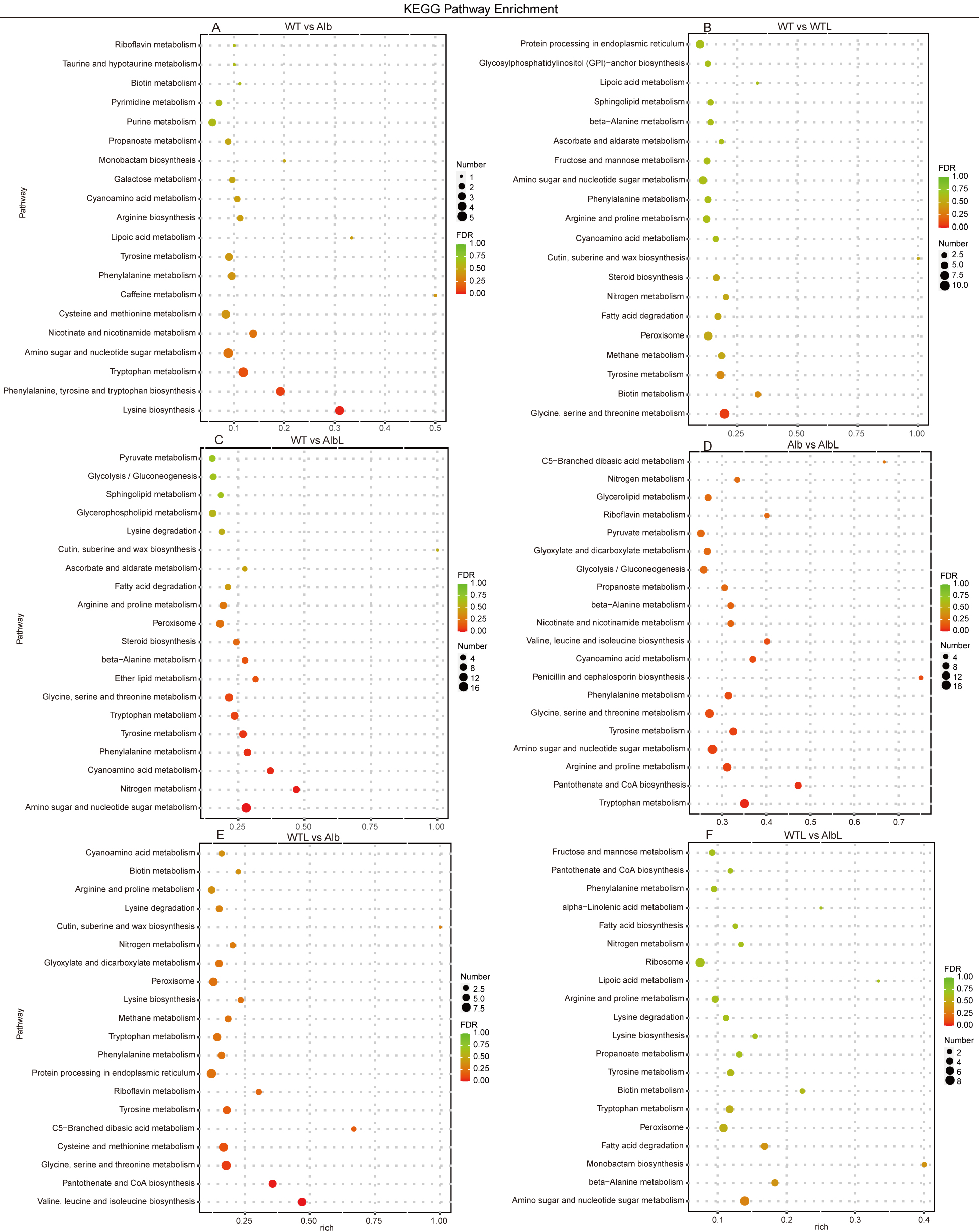

Supplement: Supplementary Figure 2 — The KEGG pathway enrichment analysis of the WT and Alb samples before and after light treatment. (A–F) Respectively indicates the KEGG pathway enrichment in WT vs. Alb, WT vs. WTL, WT vs. AlbL, Alb vs. AlbL. WTL vs. Alb, and WTL vs. AlbL. [file Image_2.JPEG]

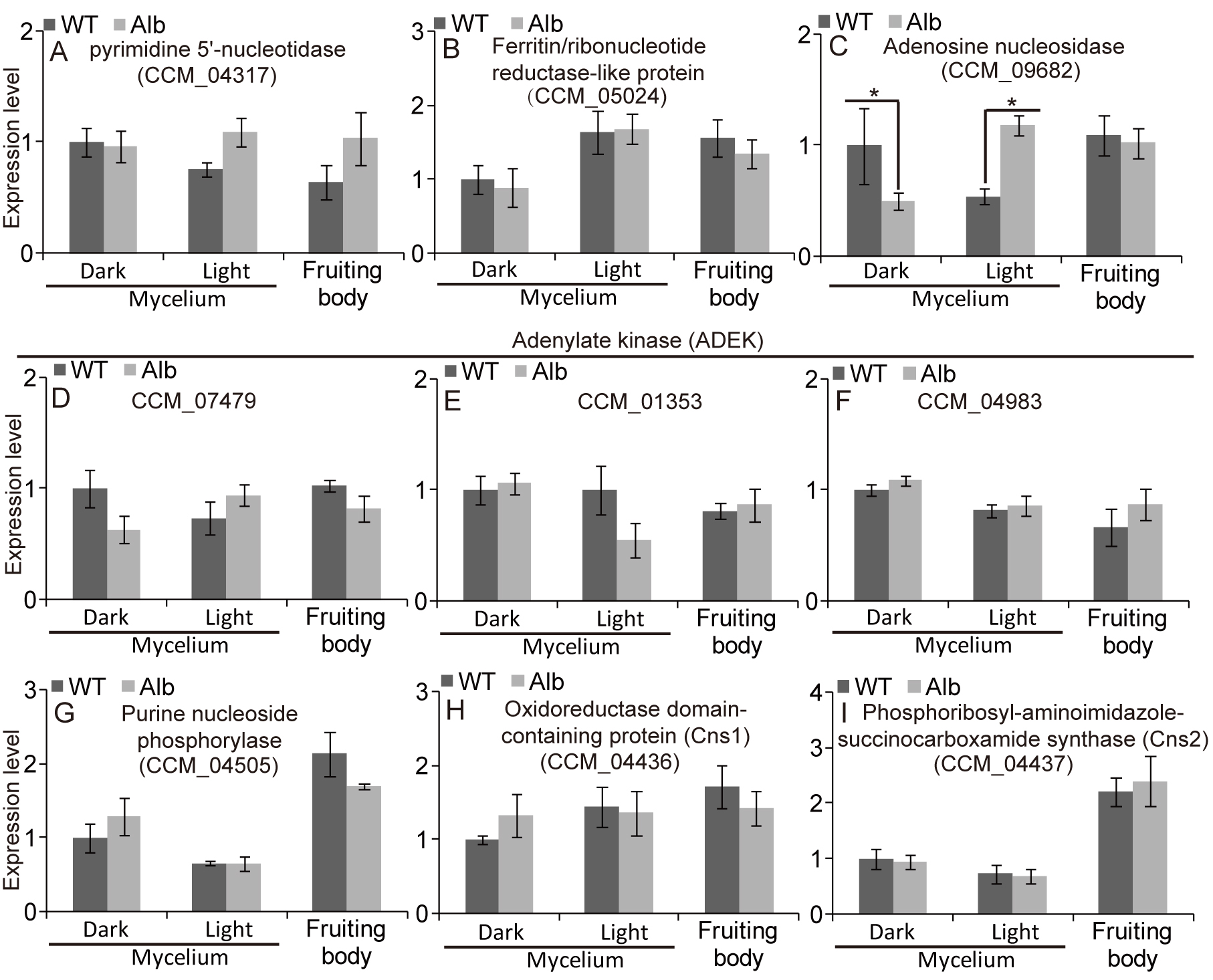

Supplement: Supplementary Figure 3 — Expression analysis of cordycepin biosynthesis genes in WT and Alb. (A–I) Respectively indicates the expression levels of pyrimidine 5′-nucleotidase (CCM_04317), ferritin/ribonucleotide reductase-like protein (CCM_05024), Adenosine nucleosidase (CCM_09682), adenylate kinase (CCM_07479, CCM_01353, and CCM_04983), purine nucleoside phosphorylase (CCM_04505), oxidoreductase domain- containing protein (Cns1) (CCM_04436), and phosphoribosyl-aminoimidazole-succinocarboxamide synthase (Cns2) (CCM_04437). One asterisks denote significance relative to WT (Student’s t-test; *p < 0.05). [file Image_3.JPEG]

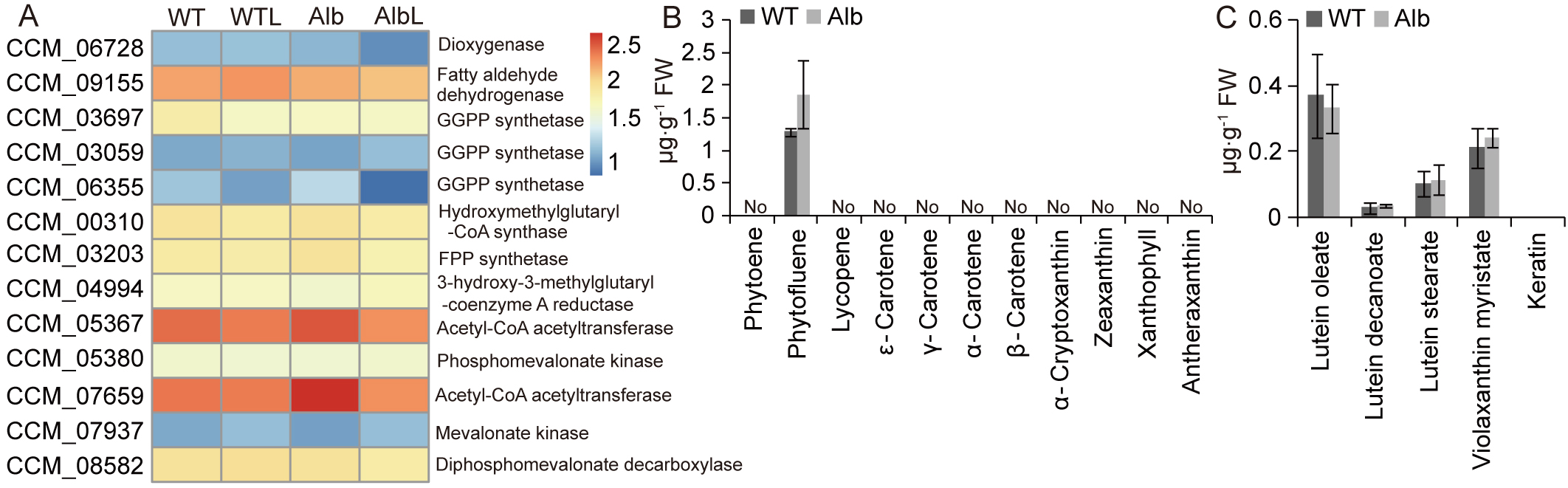

Supplement: Supplementary Figure 4 — Analysis of the products and related gene expression in carotenoid biosynthesis and metabolism. (A) Heatmap analysis of dioxygenase (CCM_06728), fatty aldehyde dehydrogenase (CCM_09155), GGPP synthetase (CCM_03697, CCM_03059, and CCM_06355), hydroxymethylglutaryl-CoA synthase (CCM_00310), FPP synthetase (CCM_03203), 3-hydroxy-3-methylglutaryl-coenzyme A reductase (CCM_04994), acetyl-CoA acetyltransferase (CCM_05367), phosphomevalonate kinase (CCM_05380), acetyl-CoA acetyltransferase (CCM_07659), mevalonate kinase (CCM_07937), and diphosphomevalonate decarboxylase (CCM_08582) in the mycelia and fruiting body of WT and Alb before and after light treatment. (B,C) Respectively indicated the product contents of carotenoid biosynthesis and metabolism. [file Image_4.JPEG]
